# Comparative characterization of lung and brain pericytes reveals tissue-specific marker and mesenchymal signatures

**DOI:** 10.3389/fcell.2026.1950106

**Published:** 2026-10-30

**Authors:** Isabel María Martínez, Antonio Martínez-Sanchez, Miguel Blanquer, Rut Valdor

**Affiliations:** 1 Biochemistry, Molecular Biology B, and Immunology Department, University of Murcia (UMU), Murcia, Spain; 2 Unit of Autophagy, Immune Response and Tolerance in Pathologic Processes, Biomedical Research Institute of Murcia-Pascual Parrilla (IMIB), Murcia, Spain; 3 Cell Therapy and Hematopoietic Transplant Group-Internal Medicine Department, UMU, Murcia, Spain; 4 Virgen de La Arrixaca University Hospital, Cell Therapy and SCT Group, IMIB, Murcia, Spain

**Keywords:** brain, CD146, lung, mesenchymal identity, mesenchymal stromal cells, pericyte heterogeneity, pericytes, RGS5

## Abstract

**Background:**

Pericytes are perivascular cells that contribute to vascular homeostasis, tissue repair, and microenvironmental regulation. Increasing evidence suggests that pericytes share phenotypic and functional features with mesenchymal stromal cells (MSCs), although the extent of this relationship and its conservation across tissues remain poorly understood. In particular, whether distinct pericyte subpopulations exhibit differential mesenchymal characteristics depending on tissue context has not been systematically investigated.

**Methods:**

CD146-positive and CD146-negative pericyte populations were isolated from murine brain and lung microvasculature using magnetic cell sorting. Canonical pericyte markers (PDGFRβ, RGS5, NG2 and α-SMA), endothelial and hematopoietic markers (CD31, CD34 and CD45), and mesenchymal-associated markers (CD90, CD44 and CD105) were analyzed by multiparametric flow cytometry to compare pericyte subsets across tissues.

**Results:**

CD146 identified distinct non-endothelial pericyte populations in both brain and lung tissues. In both organs, CD146^+^ pericytes exhibited increased expression of mesenchymal-associated markers compared with their CD146^-^ counterparts, supporting the presence of a conserved mesenchymal-like pericyte compartment. However, the organization of this phenotype differed between tissues. Brain-derived pericytes displayed significantly higher expression of RGS5, α-SMA and NG2 than lung-derived pericytes, indicating tissue-specific differences in canonical pericyte identity. Within the mesenchymal-associated compartment, CD90 emerged as the principal marker distinguishing CD146^+^ pericytes, whereas CD44 and CD105 remained broadly expressed across pericyte populations from both tissues.

**Conclusion:**

Our findings support the concept that pericytes constitute a heterogeneous component of the mesenchymal stromal cell compartment while preserving a conserved mesenchymal-associated core across tissues. Comparative analysis of brain- and lung-derived pericytes revealed that, despite sharing key mesenchymal-associated characteristics, these populations differ in the organization of both canonical pericyte markers and mesenchymal-associated features. These findings provide new insight into pericyte heterogeneity and establish a comparative framework for investigating how tissue-specific pericyte states contribute to tissue homeostasis and disease.

## 1 Introduction

Pericytes (PCs) are mural cells embedded within the microvascular wall that play essential roles in vascular stabilization, angiogenesis, blood flow regulation, and tissue homeostasis (Armulik et al., 2011; Sweeney et al., 2016). Beyond their classical vascular functions, accumulating evidence indicates that PCs actively participate in the regulation of local microenvironments through extracellular matrix remodeling, direct cell-cell interactions, and paracrine signaling (Yamazaki and Mukouyama, 2018; Valdor et al., 2019; Salinas and Valdor, 2022; Klouda et al., 2025; Salinas et al., 2026 a; Salinas et al., 2026b). However, PCs do not constitute a uniform cell population. Instead, they display remarkable heterogeneity across organs and vascular beds, differing in abundance, molecular identity, and functional properties according to their tissue of residence (Bohannon et al., 2021; Pfau et al., 2024; van Splunder et al., 2024). This diversity is reflected by the absence of a universal pericyte marker and by the use of combinations of markers such as PDGFRβ, NG2/CSPG4, RGS5, CD146, NOTCH3, and α-smooth muscle actin (α-SMA) to identify distinct pericyte subpopulations (Pfau et al., 2024; van Splunder et al., 2024). Importantly, accumulating evidence indicates that pericyte subpopulations defined by distinct marker combinations may exhibit different biological properties and functional specializations (Bohannon et al., 2021; Loan et al., 2024; Buizza et al., 2025).

The heterogeneity observed among pericyte populations is thought to arise not only from local microenvironmental cues but also from differences in developmental origin (Yamazaki and Mukouyama, 2018). Importantly, pericyte heterogeneity is not restricted to differences between organs, as pericytes of distinct developmental origins may coexist within the same tissue, further contributing to functional diversity (Prazeres et al., 2017; 2018; Pfau et al., 2024; van Splunder et al., 2024). Lineage-tracing studies have demonstrated that pericytes derive from multiple embryonic sources depending on their anatomical location. While pulmonary pericytes originate predominantly from mesodermal progenitors, brain pericytes arise from both mesodermal and neural crest-derived lineages, with neural crest contributions being particularly prominent in the forebrain vasculature (Etchevers et al., 2001; Faal et al., 2019).

These ontogenetic differences suggest that developmental history may contribute to tissue-specific molecular programs and influence the functional specialization of pericyte populations across organs (Yamazaki and Mukouyama, 2018; van Splunder et al., 2024). Notably, recent single-cell and spatial profiling studies have revealed substantial heterogeneity not only between organs but also between vascular territories within the same tissue, indicating that pericyte identity is shaped by a combination of developmental origin and local environmental cues (Pfau et al., 2024; Loan et al., 2024).

In parallel with their developmental diversity, pericytes have attracted considerable attention as potential mesenchymal stromal cell (MSC)-like populations (van Splunder et al., 2024; Salinas et al., 2026a). Seminal studies identified CD146+ perivascular cells, including pericytes, as an important source of mesenchymal stromal progenitors in multiple adult tissues, establishing a direct link between the vascular niche and mesenchymal stem cell identity (Crisan et al., 2008; Crisan et al., 2012). Beyond its use as a pericyte marker, CD146 functions as an adhesion receptor that regulates endothelial-pericyte communication and vascular stability. In the brain, CD146 coordinates endothelial cell-pericyte interactions required for blood–brain barrier formation and maturation, highlighting its functional role in neurovascular homeostasis (Chen et al., 2017). These observations suggest that CD146 expression may identify pericyte populations involved not only in vascular stabilization but also in microenvironmental communication and tissue remodeling (Chen et al., 2017). In agreement with this view, CD146 has been reported to identify mesenchymal stromal cell subsets with enhanced immunomodulatory and regenerative properties, supporting the notion that CD146+ pericytes may represent a functionally distinct population with increased mesenchymal potential (Bikorimana et al., 2022; Wu et al., 2016).

Consistent with this concept, markers such as CD90, CD44, and CD105 have been associated with mesenchymal identity, tissue regeneration, and immunomodulatory functions (Dominici et al., 2006; Soliman et al., 2021). However, the extent to which all pericytes possess equivalent stem cell properties remains controversial (Guimarães-Camboa et al., 2017). Increasing evidence suggests that pericytes exhibit varying degrees of mesenchymal plasticity and regenerative potential depending on tissue context and physiological state (Prazeres et al., 2017; Gaceb et al., 2018; Geranmayeh et al., 2019; Zhu et al., 2022; van Splunder et al., 2024). Consistent with this concept, recent work demonstrated that maintenance of pericyte stem-like properties is required for brain tissue homeostasis, further supporting the existence of functionally distinct pericyte states within the adult brain (Salinas et al., 2026a). Accordingly, differences in mesenchymal-associated marker expression may reflect distinct functional states rather than fixed cellular identities.

Among the various organs containing specialized pericyte populations, the brain and lung represent two particularly distinct microvascular environments with markedly different physiological requirements. Brain pericytes are integral components of the neurovascular unit and play essential roles in blood–brain barrier maintenance, regulation of cerebral blood flow, immune surveillance, and tissue repair (Armulik et al., 2011; Sweeney et al., 2016; van Splunder et al., 2024; Roedel et al., 2025). In addition, increasing evidence indicates that brain pericytes actively modulate local microenvironments through secreted factors and interactions with resident neural and immune cells (Yamazaki and Mukouyama, 2018; Valdor et al., 2019; Molina et al., 2022; Buizza et al., 2025; Salinas et al., 2026a;b). Their functional heterogeneity has become increasingly apparent in recent years. Distinct brain pericyte subsets contribute differentially to extracellular matrix remodeling, neurovascular repair, inflammatory regulation, and tissue regeneration (Bohannon et al., 2021; van Splunder et al., 2024). Furthermore, single-cell analyses have identified pericyte populations with distinct transcriptional profiles and reprogramming capacities following injury, highlighting the remarkable plasticity of these cells (Loan et al., 2024). This functional plasticity is further illustrated in glioblastoma, where pericytes located at different distances from the tumor exhibit distinct immune regulatory properties, emphasizing the dynamic influence of local microenvironments on pericyte behavior (Buizza et al., 2025). These observations support the concept that marker-defined pericyte subpopulations may possess distinct biological functions depending on their tissue context and local environment.

In contrast, pulmonary pericytes reside within a vascular network specialized for efficient gas exchange and continuously exposed to mechanical stress, environmental insults, and inflammatory stimuli Within this environment, they contribute to alveolar-capillary integrity, endothelial barrier regulation, angiogenesis, and tissue repair (Yuan et al., 2019; Klouda et al., 2025). Pulmonary pericytes also participate actively in inflammatory responses by sensing cytokines, regulating leukocyte trafficking, and interacting with innate immune pathways (Rayner et al., 2023; Klouda et al., 2025). Under pathological conditions, including pulmonary fibrosis, pulmonary hypertension, chronic inflammatory lung diseases, and acute respiratory distress syndrome, pulmonary pericytes exhibit substantial phenotypic plasticity and adopt activated states associated with vascular remodeling, extracellular matrix deposition, inflammatory signaling, and altered tissue repair responses (Yuan et al., 2021; Khan et al., 2022; Rayner et al., 2023; Klouda et al., 2025).

Together, these findings indicate that pulmonary pericytes are active regulators of inflammatory and remodeling responses rather than passive vascular support cells. Collectively, these studies indicate that pericyte identity is shaped by a combination of developmental origin, tissue-specific microenvironmental cues, and dynamic responses to physiological and pathological stimuli. Despite growing recognition of organ-specific pericyte heterogeneity, direct comparative analyses of brain- and lung-derived pericytes using equivalent isolation and characterization strategies remain limited. Furthermore, the extent to which developmental origin and tissue-specific environments influence mesenchymal-associated phenotypes within defined pericyte populations remains poorly understood.

In the present study, we isolated CD146+ perivascular cells from murine brain and lung tissues and systematically characterized their expression of canonical pericyte markers, including PDGFRβ, NG2, RGS5, and α-SMA. We subsequently examined the expression of mesenchymal-associated markers, including CD90, CD44, and CD105, to determine whether brain and lung pericytes share common mesenchymal features or display tissue-specific phenotypic signatures. By defining similarities and differences between these two pericyte populations, this work provides new insight into the relationship between developmental origin, pericyte heterogeneity and mesenchymal identity, while establishing a framework for understanding how tissue-specific pericyte states may differentially contribute to inflammatory, regenerative and tumor-associated microenvironments.

## 2 Materials and methods

### 2.1 Mice

Male and female mice of six to eight weeks-old C57BL/6 (Charles River Laboratories) were maintained under specific pathogen-free (SPF) conditions in the animal facilities of the University of Murcia and of the Institute of Biomedical Research of Murcia (IMIB). All animal procedures were approved and performed according to the guidelines set by the Institutional Animal Care and Use Committee of the University of Murcia (approved protocol A13150201) and of the IMIB (approved protocol A13210202).

### 2.2 Isolation and CD146+ enrichment of brain- and lung-derived pericytes

Primary murine PCs were isolated from microvasculature of brain (PCBs) and lung (PCLTs). Both PCs were isolated according to the method of Oishi et al. (2007), as described previously for PCBs (Valdor et al., 2017; Salinas et al., 2026a). Brains (excluding the cerebellum, brainstem, meninges and olfactory bulb) and whole lungs obtained from a pool of 4–6 male and female mice were mechanically dissociated and incubated in Dulbecco’s modified Eagle’s medium (DMEM; Biowest, L0101) containing 1 mg/mL collagenase A (Roche, 10103578001) and 15 μg/mL DNase I (Roche, 10104159001) for 30 min at 37 °C with gentle agitation. The digestion was stopped by dilution with culture medium followed by centrifugation. After centrifugation and washing, the microvessel-enriched fraction was collected and cultured to allow expansion until passage 5.

Both PCs were cultured in Minimum Essential Medium Alpha (MEM-α; Gibco, 15430584) supplemented with 20% (v/v) fetal bovine serum (FBS; Cytiva, 12389802), 100 U/mL penicillin–streptomycin (Corning, 30-002-CI), and 2 mM GlutaMAX (Gibco, 35050061) at 37 °C in a humidified incubator with 8.5% CO_2_.

Magnetic-activated cell sorting (MACS) was performed at passage 5 as an additional enrichment step for CD146+ cells from brain- and lung-derived microvascular cultures. Briefly, cells were harvested and resuspended in 45 µL of fluorescence-activated cell sorting buffer (FACS buffer), consisting of phosphate-buffered saline (PBS; Gibco, 14190-094) supplemented with 0.5% FBS, 2 mM ethylenediaminetetraacetic acid (EDTA; Invitrogen, 15575-038), and 1% penicillin–streptomycin (Corning, 30-002-CI). Cell suspensions were incubated with CD146 MicroBeads (Miltenyi Biotec, 130-092-007) according to the manufacturer’s instructions and subsequently passed through a magnetic separation column placed in a MidiMACS Separator (Miltenyi Biotec, 130-042-301). The retained CD146+ fraction was collected and used directly for subsequent phenotypic characterization, without further expansion.¨

### 2.3 Immunophenotyping by Flow cytometry

Immunophenotyping of CD146+ and CD146- PCBs and PCLTs was performed by multiparametric flow cytometry at sixth passage. Cells were first stained with Viability 405/520 fixable Dye (1:1000; Miltenyi Biotec, 130-130404) diluted in FACS Buffer. Fc receptors were blocked using anti-mouse CD16/CD32 antibody (1:100, Invitrogen, 14-0161-85). Cells were then stained for extracellular markers using the following antibodies diluted in FACS Buffer for 20 min at 4 °C: CD146-APC (1:100; Miltenyi Biotec, 130-118-408), CD140b-PE (1:100; PDGFRβ; Miltenyi Biotec, 130-123-271). MSC marker expression was analyzed with the following anti-mouse antibodies: CD44-APC-Cy7 (1:100; BD biosciences, 560568), CD105-PE-Cy7 (1:100; Miltenyi Biotec, 130-118-177), and CD90.2-BV421 (1:100; Miltenyi Biotec, 130–102–345). Following extracellular staining, cells were fixed with 2% paraformaldehyde (PFA) solution for 15 min at 4 °C. This working solution was freshly diluted in PBS from a 20% stock prepared from powder (Sigma-Aldrich, P6148). Cells were permeabilized using 0.1% Tween-20 (Sigma-Aldrich, P1379) in FACS Buffer for 15 min at 4 °C. Intracellular staining was performed for 20 min at 4 °C using antibodies diluted in FACS Buffer against RGS5 (1:100; Invitrogen, PA5101835), AN2-A488 (1:100; Sigma-Aldrich, AB5320A4) and α-SMA (1:100; Abcam, ab7817). Finally, cells were incubated with the corresponding secondary antibodies for 20 min at 4 °C, anti-rabbit-A488 (1:200; Invitrogen, A21206) for RGS5 detection and anti-mouse-PERCP (1:200; 130-103-044) for α-SMA detection.

To ensure comprehensive characterization and prevent fluorochrome overlap, the aforementioned markers were distributed into two distinct multiparametric panels. Panel one consisted of a mixture evaluating CD146, α-SMA, PDGFRβ, AN2 (NG2), CD90.2, CD105, and CD44. Panel two was designed to evaluate CD146, α-SMA, PDGFRβ, RGS5, CD90.2, CD105, and CD44. For multicolor flow cytometry, fluorescence compensation was established using single-stained controls for each fluorochrome included in the panel to correct for spectral overlap between detection channels. The resulting compensation settings were incorporated into the acquisition protocol and consistently applied across experiments, with periodic verification using single-stained controls. To ensure accurate data interpretation, establish precise gating boundaries, and control for non-specific binding, matched fluorochrome-conjugated isotype controls were included for all directly stained markers. The specific isotype controls utilized were: APC (1:100, Miltenyi Biotec, 130-113-446), PE (1:100, Miltenyi Biotec, 130-113-450), APC-Vio770 (1:100, Miltenyi Biotec, 130-113-447), PE-Vio770 (1:100, Miltenyi Biotec, 130-113-452), and VioBlue (1:100, Miltenyi Biotec, 130-103-083). For intracellular markers evaluated via indirect staining (RGS5 and α-SMA), non-specific background was controlled by including secondary antibody-only controls (omitting the primary antibodies). Labelled cells were analyzed by flow cytometry using a BD LSR Fortessa X-20 flow cytometer (BD Biosciences) and data were analyzed with FlowJo software (Ver. 10.0.7, TreeStar). Please, see Supplementary Figure S1 for the detailed sequential gating strategy. All determinations were performed on at least three independent biological cell cultures (each derived from a distinct pool of animals), with technical replicates analyzed for each independent experiment.

Additionally, a third independent flow cytometry panel was performed to confirm the absence of endothelial and hematopoietic contamination in the isolated pericyte populations. Detailed methodology and the corresponding gating strategy for this purity assessment are provided in the Supplementary Methods and Supplementary Figure S2.

### 2.4 Statistical analysis

Differences between grouped variables were analyzed by two-way ANOVA followed by Sidak multiple comparisons post-test. Statistical significance was defined as p < 0.05 and indicated as *p < 0.05, **p < 0.01, ***p < 0.001, ****p < 0.0001 or ns for no significance. Analysis was done with GraphPad Prism (version 8.3.0).

## 3 Results

### 3.1 CD146 defines distinct pericyte subpopulations within the brain and lung microvasculature

In a previous study, our group characterized the pericyte population within the brain microvasculature (Salinas et al., 2026a). Among the markers analyzed, CD146 was of particular interest, as it has been widely recognized as a pericyte marker across multiple tissues, including the brain, adipose tissue, and lung. Furthermore, CD146 expression has been associated with perivascular cell populations exhibiting enhanced mesenchymal and regenerative potential (Crisan et al., 2012; Wangler et al., 2019; Bikorimana et al., 2022). While brain-derived pericytes have been proposed as tissue-resident MSC-like cells with therapeutic potential, the mesenchymal characteristics and regenerative potential of lung-derived pericytes remain largely unexplored. Importantly, brain and lung pericytes arise from distinct developmental contexts. Whereas pulmonary pericytes are thought to derive predominantly from mesodermal progenitors, brain pericytes comprise populations originating from both mesodermal and neural crest lineages depending on their anatomical location (Prazeres et al., 2017; Yamazaki and Mukouyama, 2018). These developmental differences may contribute to tissue-specific phenotypic and functional specialization. Therefore, we sought to characterize pericytes within the lung stromal vascular fraction (SVF) and compare their mesenchymal features with those of brain-derived pericytes to determine the extent to which both populations share a conserved MSC-like phenotype.

MACS-based CD146 selection was performed on brain (PCBs) and lung (PCLTs) microvasculature cell populations (Figure 1A). Subsequently, CD146^+^ and CD146^-^ subsets were analyzed for canonical pericyte markers by flow cytometry. We confirmed that a large proportion of CD146^+^ PCBs co-expressed PDGFRβ and RGS5 (∼83%), while approximately 52% were double-positive for PDGFRβ and α-SMA (Figure 1B). Mean fluorescence intensity (MFI) analysis further confirmed the strong expression of PDGFRβ, RGS5 and α-SMA, whereas NG2 expression was comparatively lower (∼24%). In contrast, the CD146^-^ subset exhibited a markedly reduced expression profile of pericyte markers: ∼36% co-expressed PDGFRβ and RGS5, ∼14% were double-positive for PDGFRβ and α-SMA, and only ∼6% co-expressed PDGFRβ and NG2 (Figure 1C). Quantitative analysis corroborated that the proportion of positive cells for all evaluated canonical markers was significantly higher in the CD146^+^ population than in the CD146^-^ counterpart (Figure 1D), a trend similarly reflected in the significantly higher MFI levels across all markers for the CD146^+^ fraction (Figure 1E).

**FIGURE 1:**
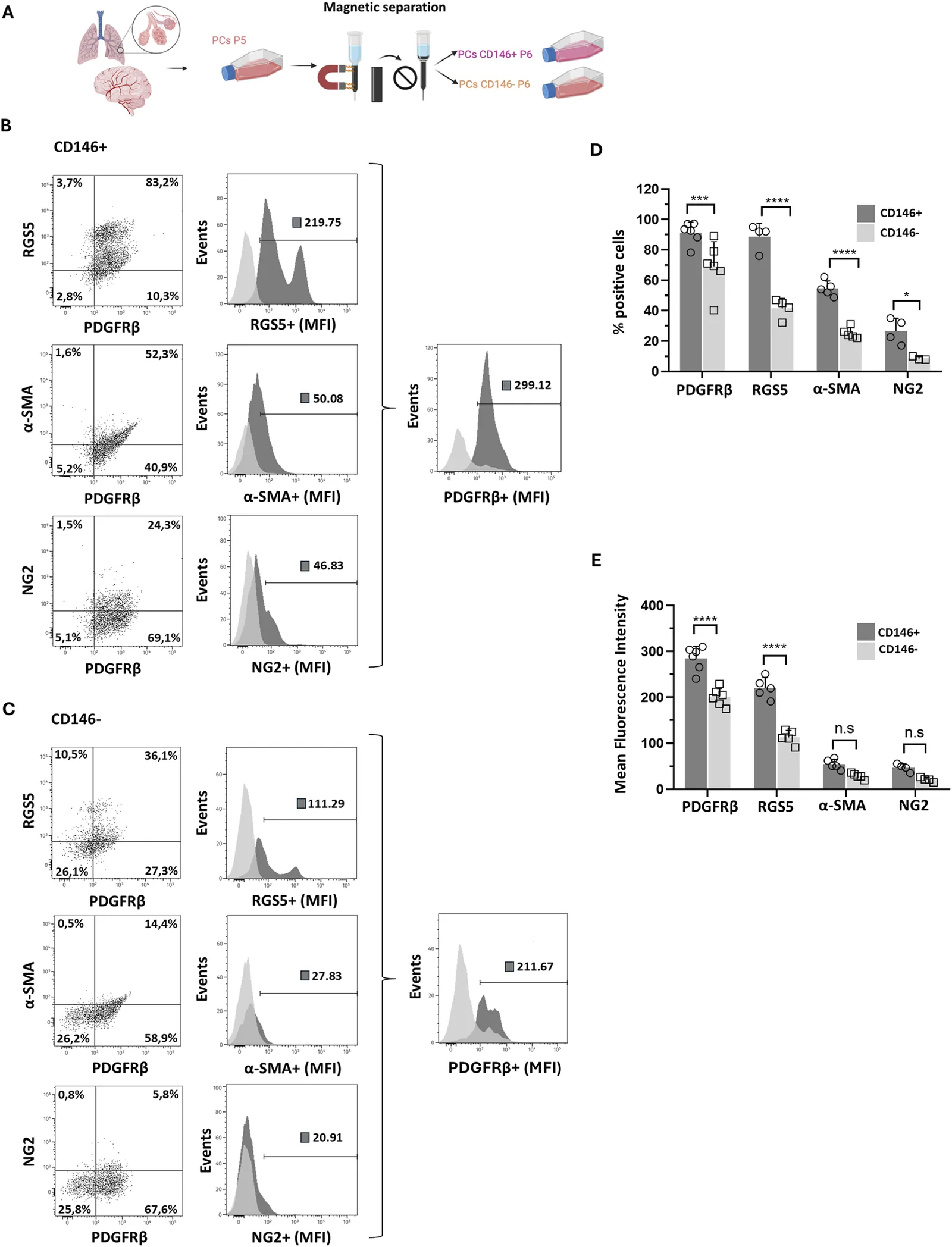
CD146-Based Enrichment Reveals Comparable Pericyte Subpopulations in Brain Tissue. **(A)** Schematic overview of pericyte isolation from brain and lung tissues followed by CD146-based magnetic separation. **(B, C)** Flow cytometry analysis of pericyte marker expression (NG2, PDGFRβ, RGS5, and α-SMA) in primary brain-derived pericytes (PCBs) after magnetic separation into CD146^+^ **(B)** and CD146^-^ **(C)** fractions. Data are expressed as mean fluorescence intensity (MFI). Nonspecific fluorescence was assessed using isotype-matched control antibodies (light grey). **(D)** Comparative analysis of canonical pericyte marker expression between brain-derived pericyte CD146^+^ and CD146^-^ fractions. Bar graphs represent the percentage of positive cells. **(E)** Comparative analysis of canonical pericyte marker expression between brain-derived pericyte CD146^+^ and CD146^-^ fractions. Bar graphs represent the mean fluorescence intensity (MFI). Data are presented as mean ± SD, where individual data points (dots) represent technical replicates obtained across a minimum of three independent experiments, each generated from a different pool of 4–6 mice. Statistical significance was determined using a two-way ANOVA followed by Sidak’s multiple comparisons test: *p < 0.05; p < 0.01; ***p < 0.001; ****p < 0.0001; n. s., not significant.

Next, canonical pericyte marker expression was evaluated in CD146^+^ and CD146^-^ PCLTs. Similar to brain-derived pericytes, the CD146^+^ pulmonary population showed high co-expression of PDGFRβ and RGS5 (∼73%), whereas PDGFRβ co-expression with α-SMA and NG2 was markedly lower (∼19% and ∼6%, respectively) (Figure 2A). MFI analysis confirmed strong PDGFRβ and RGS5 expression together with comparatively lower α-SMA and NG2 expression. In contrast, the CD146^-^ pulmonary fraction displayed an overall reduction in canonical pericyte markers, with approximately 49% of cells co-expressing PDGFRβ and RGS5, ∼14% co-expressing PDGFRβ and α-SMA and only ∼5% co-expressing PDGFRβ and NG2 (Figure 2B). Quantitative analysis confirmed that the proportions of PDGFRβ^+^, RGS5^+^ and α-SMA^+^ cells were significantly higher in the CD146^+^ pulmonary fraction, whereas NG2 expression remained low and did not differ significantly between CD146^+^ and CD146^-^ PCLTs (Figure 2C), a pattern consistent with the MFI levels observed across these subsets (Figure 2D).

**FIGURE 2:**
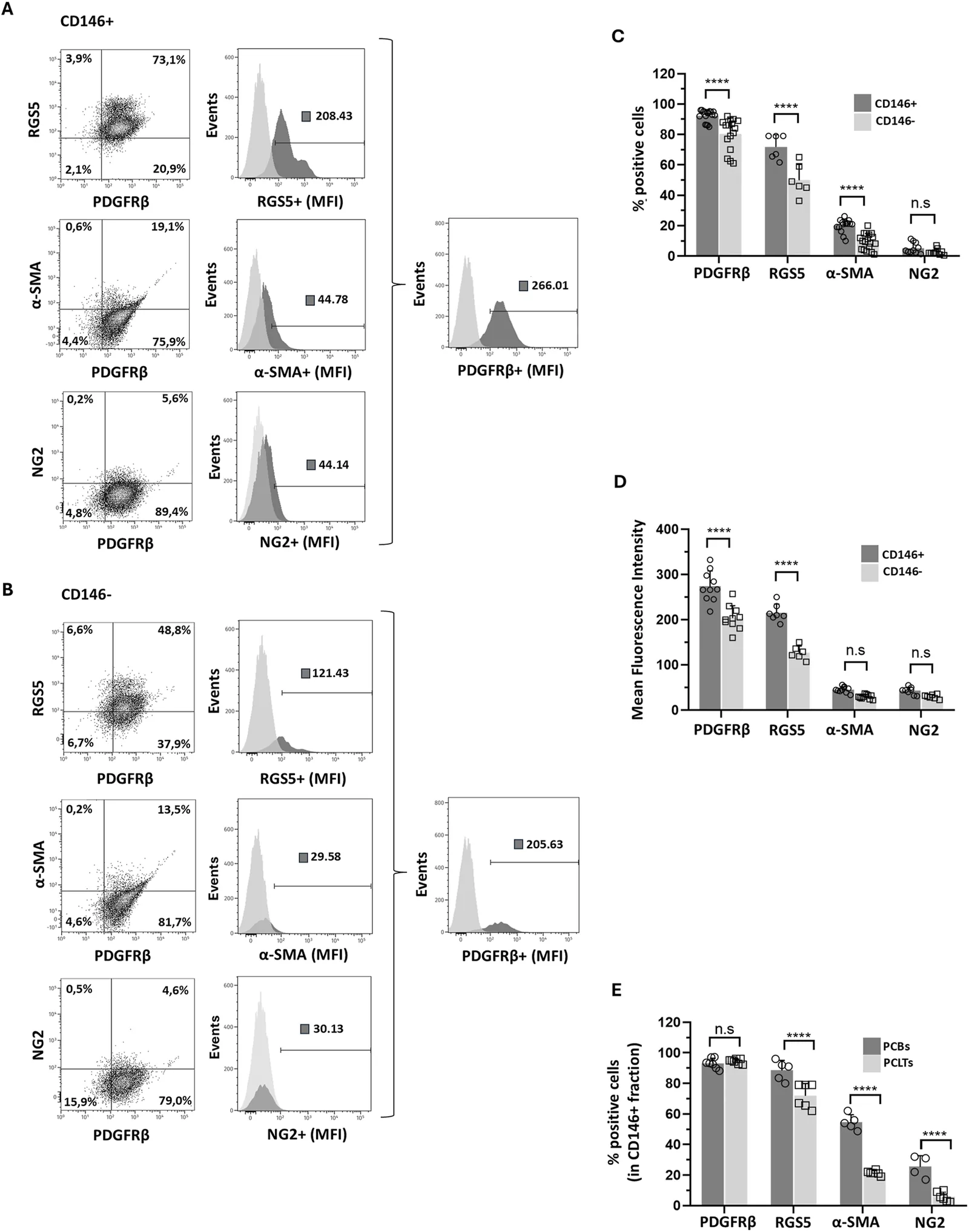
CD146-Based Enrichment Reveals Comparable Pericyte Subpopulations in Lung Tissue. **(A, B)** Flow cytometry analysis of pericyte marker expression (NG2, PDGFRβ, RGS5, and α-SMA) in primary lung PCs (PCLTs) after magnetic separation into CD146^+^ **(A)** and CD146^-^ **(B)** fractions. Data are expressed as mean fluorescence intensity (MFI). Nonspecific fluorescence was assessed using isotype-matched control antibodies. **(C)** Comparative analysis of canonical pericyte marker expression between brain-derived pericyte CD146^+^ and CD146^-^ fractions. Bar graphs represent the percentage of positive cells. **(D)** Comparative analysis of canonical pericyte marker expression between brain-derived pericyte CD146^+^ and CD146^-^ fractions. Bar graphs represent the mean fluorescence intensity (MFI). **(E)** Comparative analysis of canonical pericyte marker expression between brain-derived and lung-derived CD146^+^ pericyte populations. Bar graphs represent the percentage of positive cells. Data are presented as mean ± SD, where individual data points (dots) represent technical replicates obtained across a minimum of three independent experiments, each generated from a different pool of 4–6 mice. Statistical significance was determined using a two-way ANOVA followed by Sidak’s multiple comparisons test: *p < 0.05; p < 0.01; ***p < 0.001; ****p < 0.0001; n. s., not significant.

Finally, to directly compare canonical pericyte marker expression between tissues, the CD146^+^ fractions isolated from brain and lung were analyzed in parallel (Figure 2E). While both populations exhibited similarly high levels of PDGFRβ expression, brain-derived pericytes displayed significantly higher proportions of RGS5^+^, α-SMA^+^ and NG2^+^ cells than lung-derived pericytes. These results demonstrate that although CD146 consistently enriches for pericytes in both tissues, the expression of canonical pericyte markers differs substantially between brain- and lung-derived pericytes, revealing tissue-specific phenotypic organization of the pericyte compartment.

### 3.2 CD146^+^ brain- and lung-derived pericytes share a conserved mesenchymal-associated phenotype with tissue-specific differences

Mesenchymal stromal cells (MSCs) are commonly characterized by the expression of markers associated with stromal identity, regenerative potential, and cellular plasticity, including CD90, CD105, and CD44 (Dominici et al., 2006; Fonseca et al., 2023). Pericytes have been reported to share key phenotypic and functional features with MSCs, including the expression of mesenchymal-associated markers and participation in tissue homeostasis and repair, supporting their proposed MSC-like properties (Birbrair et al., 2013; Geranmayeh et al., 2019; Valdor et al., 2019; Salinas et al., 2026a). Given that CD146 is expressed by both pericytes and subsets of MSCs and has been associated with enhanced mesenchymal and regenerative properties (Crisan et al., 2012; Bikorimana et al., 2022), we sought to determine whether CD146+ pericyte populations isolated from tissues of different embryonic origins also display a similar mesenchymal-associated immunophenotype. To this end, we assessed the expression of established mesenchymal markers in CD146-defined pericyte subsets from brain and lung using flow cytometry.

We first analyzed the expression of the mesenchymal-associated markers CD90, CD105 and CD44 and their co-expression with the canonical pericyte markers PDGFRβ and RGS5 within the phenotypically defined CD146^+^ and CD146^-^ brain pericyte fractions. While CD105 and CD44 expression remained consistently high across all evaluated subsets, the CD146^+^ fraction was distinguished by a significantly higher expression of CD90 than its CD146^-^ counterpart. Flow cytometric analysis further revealed that the vast majority of CD146^+^ cells co-expressed both CD105 and CD44 together with either PDGFRβ or RGS5 (Figure 3A; Supplementary Figure S2A). Quantitative analysis demonstrated that approximately 60% of both the PDGFRβ^+^ and RGS5^+^ subsets within the CD146^+^ fraction co-expressed CD90 (Figures 3C,D), whereas CD146^-^ PCBs exhibited a markedly reduced proportion of these double-positive populations (Figure 3B; Supplementary Figure S2B). Analysis restricted to the gated PDGFRβ^+^ and RGS5^+^ pericyte subsets confirmed that CD90 expression was significantly enriched in the CD146^+^ fraction, whereas CD105 and CD44 remained consistently expressed across both subsets (Figures 3C,D). Overall, the mesenchymal-associated phenotype was highly similar between the PDGFRβ^+^ and RGS5^+^ populations within the CD146^+^ brain pericyte fraction, with CD90 representing the principal marker distinguishing CD146^+^ from CD146^-^ cells.

**FIGURE 3:**
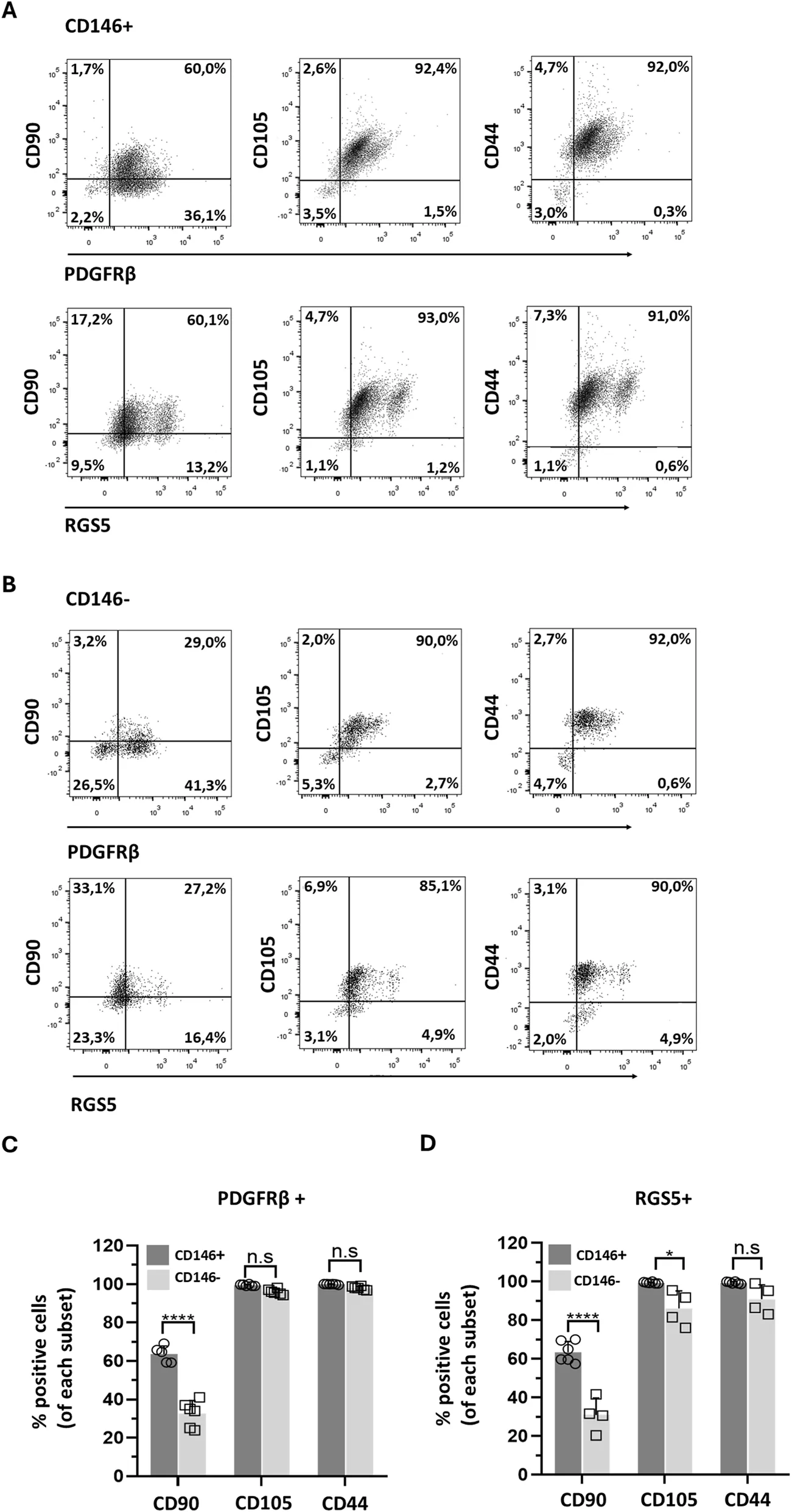
CD146^+^ Pericytes from Brain Exhibit a Conserved Mesenchymal Marker Profile. **(A, B)** Flow cytometry analysis of mesenchymal marker expression (CD90, CD105, and CD44) in primary brain-derived pericytes (PCBs) after magnetic separation into CD146^+^ **(A)** and CD146^-^ **(B)** fractions, stratified into PDGFRβ (upper dotplots) and RGS5 (lower dotplots) subpopulations. **(C, D)** Comparative analysis of mesenchymal marker expression between brain pericytes CD146^+^ and CD146^-^ fractions, stratified by PDGFRβ^+^ **(C)** and RGS5^+^ **(D)** positivity. Bar graphs represent the percentage of positive cells. Bar graphs represent the percentage of positive cells. Data are presented as mean ± SD, where individual data points (dots) represent technical replicates obtained across a minimum of three independent experiments, each generated from a different pool of 4–6 mice. Statistical significance was determined using a two-way ANOVA followed by Sidak’s multiple comparisons test: *p < 0.05; p < 0.01; ***p < 0.001; ****p < 0.0001; n. s., not significant.

We next evaluated the expression of the same mesenchymal-associated markers in pulmonary pericyte populations. Similar to brain-derived pericytes, CD146^+^ PCLTs displayed significantly higher expression of mesenchymal-associated markers than their CD146^-^ counterparts (Figures 4A,B; Supplementary Figures S3A,B). Flow cytometry demonstrated that the majority of CD146^+^ pulmonary pericytes co-expressed CD105 and CD44 together with either PDGFRβ or RGS5, whereas approximately 40% of both PDGFRβ^+^ and RGS5^+^ subsets also co-expressed CD90 (Figure 4A). Quantitative analysis confirmed that CD90, CD105 and CD44 were all significantly enriched within the CD146^+^ fraction compared with CD146^-^ pulmonary pericytes in both the PDGFRβ^+^ and RGS5^+^ subsets (Figures 4C,D). These findings indicate that CD146 effectively enriches pulmonary pericytes displaying a mesenchymal-associated phenotype characterized by the coordinated expression of the three canonical MSC-associated markers.

**FIGURE 4:**
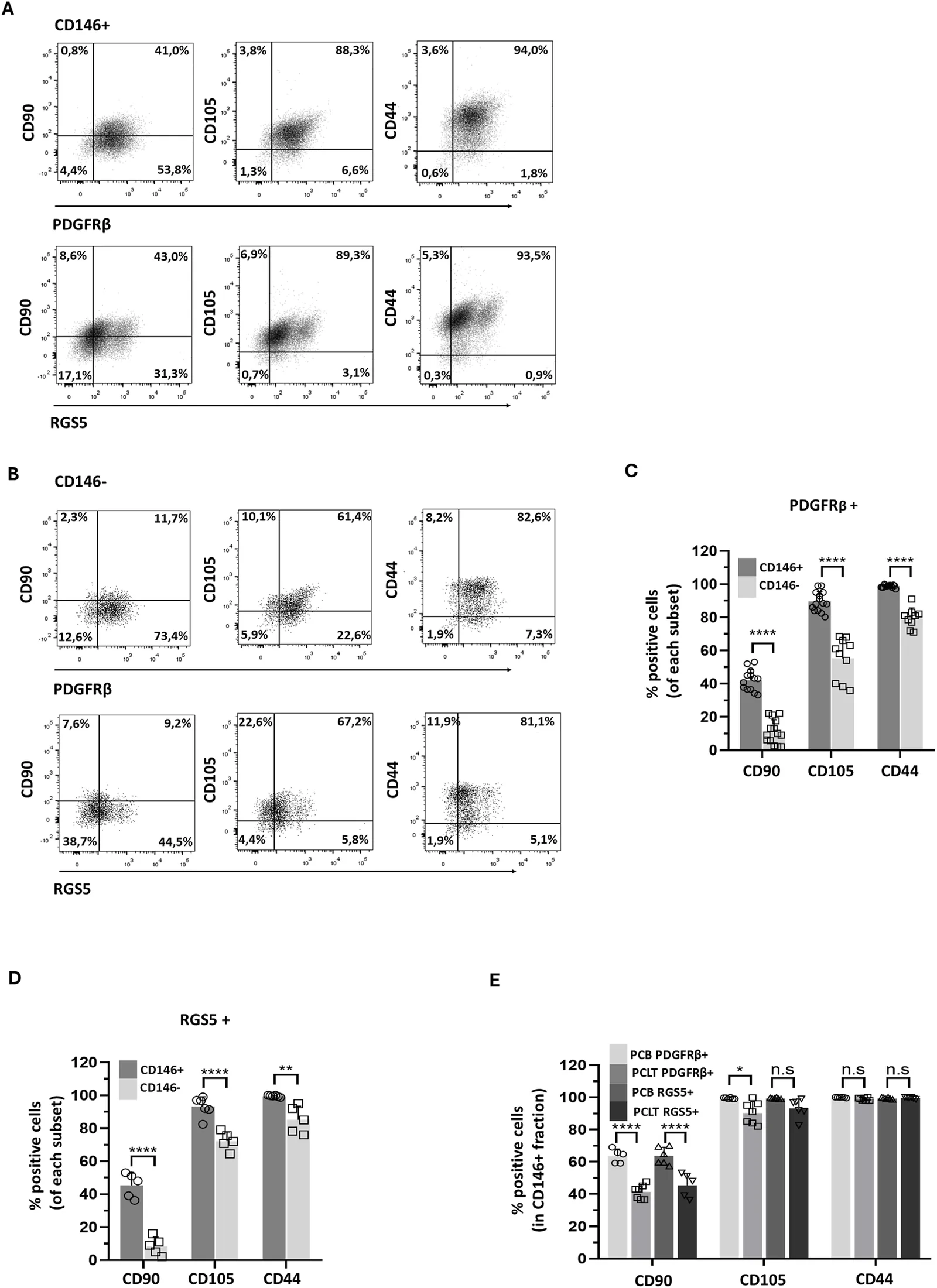
CD146^+^ Pericytes from Lung Exhibit a Partial Mesenchymal Marker Profile. **(A, B)** Flow cytometry analysis of mesenchymal marker expression (CD90, CD105, and CD44) in primary lung PCs after magnetic separation into CD146^+^ **(A)** and CD146^-^ **(B)** fractions, stratified into PDGFRβ (upper dotplots) and RGS5 (lower dotplots) subpopulations. **(C, D)** Comparative analysis of mesenchymal marker expression between lung-derived pericyte CD146^+^ and CD146^-^ fractions, stratified by PDGFRβ^+^ **(C)** and RGS5^+^ **(D)** positivity. Bar graphs represent the percentage of positive cells. **(E)** Comparative analysis of canonical mesenchymal marker expression (CD90, CD105, and CD44) across CD146^+^ pericyte subsets derived from brain and lung, stratified by PDGFRβ^+^ and RGS5^+^ positivity. Bar graphs represent the percentage of positive cells. Data are presented as mean ± SD, where individual data points (dots) represent technical replicates obtained across a minimum of three independent experiments, each generated from a different pool of 4–6 mice. Statistical significance was determined using a two-way ANOVA followed by Sidak’s multiple comparisons test: *p < 0.05; p < 0.01; ***p < 0.001; ****p < 0.0001; n. s., not significant.

Finally, we directly compared the mesenchymal-associated phenotype of CD146^+^ brain- and lung-derived pericytes by analyzing CD90, CD105 and CD44 expression within the PDGFRβ^+^ and RGS5^+^ subsets (Figure 4E). This comparative analysis demonstrated that both tissues shared similarly high CD44 expression, indicating that this marker represents a conserved mesenchymal-associated feature of CD146^+^ pericytes regardless of tissue origin. Likewise, CD105 expression remained largely comparable between brain- and lung-derived pericytes, with only a modest but significant reduction observed within the PDGFRβ^+^ pulmonary subset. In contrast, CD90 expression was consistently and significantly reduced in both PDGFRβ^+^ and RGS5^+^ pulmonary pericytes compared with their brain-derived counterparts. Together, these findings indicate that although CD146^+^ pericytes from both tissues share a conserved mesenchymal-associated phenotype, the relative organization of this phenotype differs between organs, with CD90 emerging as the principal marker distinguishing brain- and lung-derived pericytes.

Because MSC-like populations are commonly characterized not only by the expression of mesenchymal-associated markers but also by the absence of hematopoietic and endothelial markers, we further assessed the expression of CD31, CD34 and CD45. Both brain- and lung-derived CD146-defined pericyte populations exhibited negligible expression of CD31, CD34 and CD45, confirming their non-endothelial, non-hematopoietic perivascular stromal identity (Supplementary Figures S4A,B).

Finally, to determine whether this mesenchymal-associated phenotype was maintained within a more stringently phenotypically defined pericyte population, we performed a multiparametric co-expression analysis. Within the enriched CD146^+^ fractions, we utilized sequential gating to identify a triple-positive population co-expressing the conventional pericyte-associated markers PDGFRβ, α-SMA, and RGS5. We then specifically evaluated the expression of MSC-associated markers within this population displaying a robust combined pericyte-associated marker profile. Consistent with our previous observations, both brain- and lung-derived populations co-expressing these markers exhibited robust, near-ubiquitous expression of CD44 and CD105. Furthermore, the tissue-specific difference in the proportion of CD90^+^ cells was maintained within this triple-positive population (Figure 5A). Notably, when the analysis was further restricted to cells co-expressing all four pericyte-associated markers analyzed in this study (CD146, PDGFRβ, RGS5, and α-SMA), the proportion of CD90^+^ cells was higher than that observed in the previously analyzed double-positive populations, reaching approximately 80% in brain-derived pericytes and 65% in lung-derived pericytes (Figure 5B). Together, these findings demonstrate that the mesenchymal-associated phenotype, including the tissue-specific difference in CD90, is maintained within a pericyte population stringently defined by the combined expression of multiple conventional pericyte-associated markers.

**FIGURE 5:**
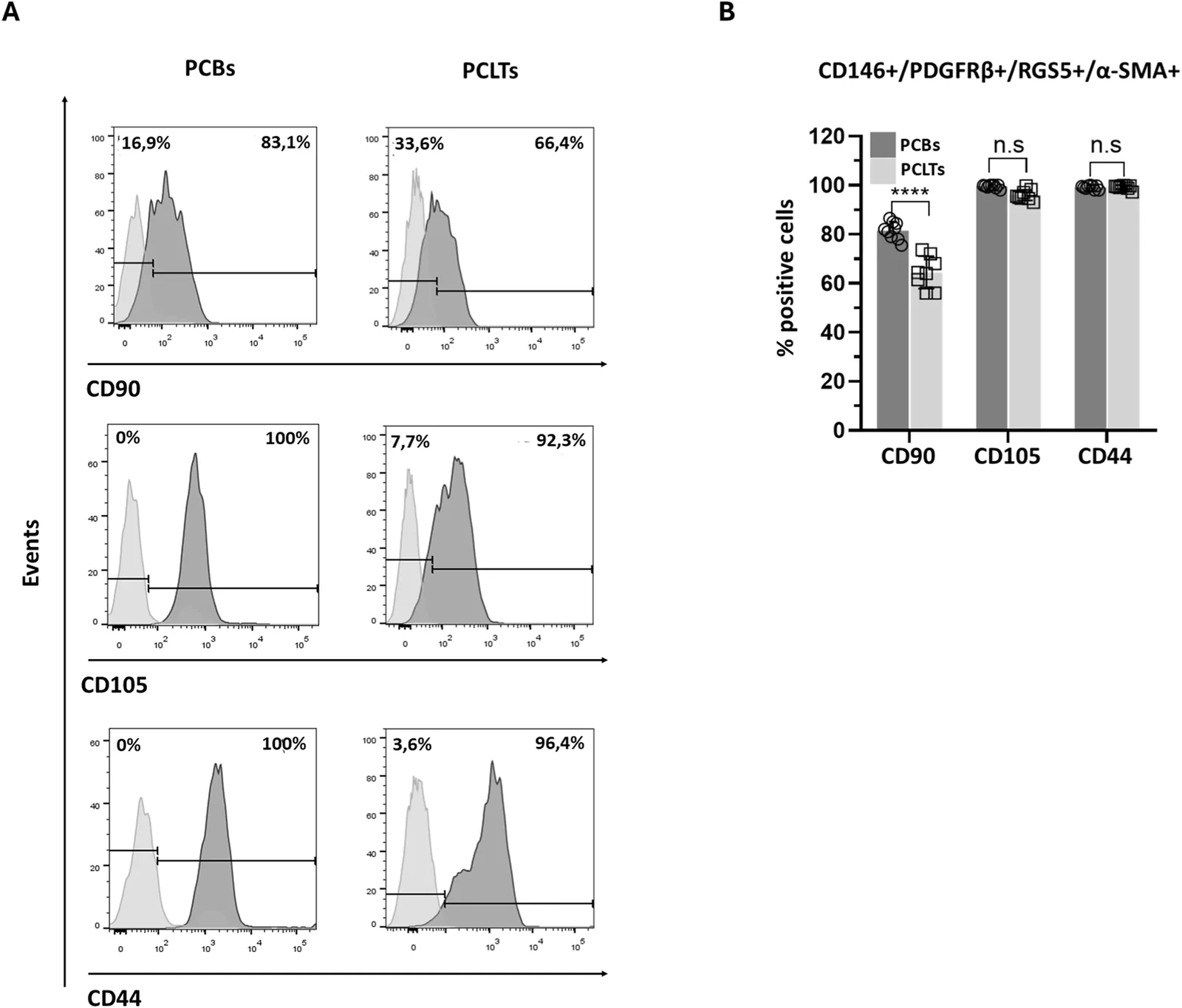
Mesenchymal-associated marker expression in the pericyte subpopulation defined by the co-expression of CD146, PDGFRβ, RGS5, and α-SMA. **(A)** Representative flow cytometry histograms showing the expression of MSC-associated markers within the quadruple-positive pericyte subpopulation (CD146^+^/PDGFRβ^+^/RGS5^+^/α-SMA^+^) derived from brain (PCBs) and lung (PCLTs) cultures. Light grey histograms represent the corresponding isotype controls, while dark grey histograms show the specific expression of CD90, CD105, and CD44. The indicated percentages represent the proportion of positive cells for each mesenchymal-associated marker within this multi-marker-positive pericyte fraction. **(B)** Quantitative analysis of the percentage of CD90^+^, CD105^+^, and CD44^+^ cells within the quadruple-positive pericyte populations from brain and lung cultures. Data are presented as mean ± SD, where individual data points (dots) represent technical replicates obtained across a minimum of three independent experiments, each generated from a different pool of 4–6 mice. Statistical significance was determined using a two-way ANOVA followed by Sidak’s multiple comparisons test: *p < 0.05; **p < 0.01; ***p < 0.001; ****p < 0.0001; n. s., not significant.

## 4 Discussion

The present study supports the growing concept that pericytes do not represent a homogeneous vascular cell population but rather comprise distinct subsets with potentially different biological functions. Using a common CD146-based isolation strategy and applying the same phenotypic characterization pipeline to two physiologically distinct organs, we identified heterogeneous pericyte populations within both the murine brain and lung microvasculature. These populations differed substantially in their expression of canonical pericyte markers and mesenchymal-associated features, indicating that pericyte identity is not restricted to a single phenotypic state. Our findings are consistent with recent studies demonstrating extensive pericyte heterogeneity across tissues, vascular territories and pathological conditions (Bohannon et al., 2021; van Splunder et al., 2024; Pfau et al., 2024). Importantly, our results extend these observations by demonstrating that phenotypic heterogeneity is retained in pericyte populations derived from both brain and lung when both tissues are analyzed using the same experimental approach. Together, these findings support the concept that pericytes comprise a spectrum of cellular states shaped by tissue-specific requirements rather than a uniform vascular support population.

Although CD146 is widely used as a pericyte marker, accumulating evidence indicates that its biological significance extends beyond cell identification. Previous studies identified CD146-positive perivascular cells as an important source of mesenchymal stromal progenitors with multilineage differentiation potential (Crisan et al., 2012; Wu et al., 2016; Wangler et al., 2019). More recently, CD146 expression has been associated with enhanced regenerative properties and tissue repair functions in perivascular stromal populations (Bikorimana et al., 2022), while also participating in endothelial–pericyte communication and vascular stabilization (Chen et al., 2017; 2018). Consistent with these observations, our results demonstrate that CD146 identifies a conserved mesenchymal-associated pericyte compartment in both brain and lung. However, rather than defining identical cell populations across tissues, CD146 reveals distinct tissue-specific organizations of this mesenchymal program. In both organs, CD146^+^ pericytes displayed enriched mesenchymal-associated features compared with their CD146^-^ counterparts, but the relative contribution of individual markers differed between tissues, indicating that the mesenchymal phenotype is organized according to the local tissue context rather than representing a uniform pericyte state.

Among the mesenchymal-associated markers analyzed, CD90 emerged as the principal feature distinguishing brain- and lung-derived pericytes. Whereas CD44 remained highly expressed in both tissues and CD105 showed only minor differences, CD90 expression was consistently higher in brain-derived pericytes. This observation is particularly relevant because CD90 is not merely a phenotypic marker but has been implicated in the regulation of stemness, proliferation and lineage commitment in mesenchymal progenitors (Moraes et al., 2016). While CD90 expression was notably reduced in pulmonary pericytes compared to their brain counterparts, our current descriptive data do not allow us to determine the functional consequences of this difference. However, considering that CD90 has been implicated in mechanosensing and the regulation of cell-matrix adhesion in mesenchymal lineages (Valdivia et al., 2023), we hypothesize that this variation might reflect a phenotypic adjustment to the distinct biomechanical properties and mechanical stress of the brain and lung microenvironments. Future functional studies will be necessary to experimentally validate this hypothesis and further elucidate the role of CD90 in pulmonary pericyte biology.

Accumulating evidence indicates that pericytes do not represent terminally differentiated vascular cells but instead occupy a spectrum of functional states with distinct regenerative, immunomodulatory and tissue-supporting capacities. Single-cell and lineage-tracing studies have demonstrated that pericyte populations differ substantially in their transcriptional programs, regenerative potential and responses to tissue injury (Loan et al., 2024; Pfau et al., 2024). Following ischemic injury, subsets of brain pericytes acquire multipotential stem cell-like properties and differentiate into neural and vascular lineages (Nakagomi et al., 2015), whereas under pathological conditions other pericyte populations may adopt immune-like phenotypes resembling microglia and macrophages (Nirwane and Yao, 2022). Consistent with this concept, disruption of pericyte homeostatic programs shifts these cells from regenerative to inflammatory states, profoundly altering their interactions with the surrounding microenvironment (Salinas et al., 2026a). Together, these observations support the interpretation that marker-defined pericyte populations represent functionally distinct cellular states rather than merely phenotypic variants.

Brain pericytes are highly heterogeneous, comprising populations of distinct developmental origin, including both mesodermal and neural crest-derived cells (Etchevers et al., 2001; Yamazaki and Mukouyama, 2018), and displaying marked phenotypic variation along the arteriole, capillary, and venule axis (Vanlandewijck et al., 2018; Hartmann et al., 2022). Furthermore, pericyte density and phenotype may vary across anatomical brain regions and cortical layers (Bernier et al., 2021). Because our isolation procedure utilized the cerebral microvasculature after exclusion of the cerebellum, meninges, olfactory bulbs, and brainstem, the cerebral population analyzed here integrates pericytes from these diverse developmental lineages and microvascular niches. In contrast, pulmonary pericytes are generally considered to derive predominantly from mesodermal progenitors (Prazeres et al., 2017), though similar spatial heterogeneity likely exists within the pulmonary vascular bed. Consequently, the differences identified between brain- and lung-derived pericytes should be interpreted as organ-associated phenotypic differences retained under standardized *ex vivo* conditions, rather than as uniform properties of all pericytes within either organ. How specific developmental lineages and vascular zones contribute to these organ-specific programs remains an important question for future studies.

A particularly interesting observation arising from this study was the marked difference in α-SMA expression between brain- and lung-derived pericytes. While α-SMA was readily detected in a substantial proportion of brain pericytes, its expression was markedly lower in pulmonary populations. This finding further supports the concept that tissue-specific pericyte identities are maintained despite the conservation of a common mesenchymal-associated core. Such differences are consistent with the growing recognition that pericyte phenotype is strongly influenced by local microenvironmental requirements and vascular specialization rather than being solely determined by a common perivascular identity (Yamazaki and Mukouyama, 2018; Pfau et al., 2024). Brain pericytes are integral components of the neurovascular unit, contributing to blood–brain barrier maintenance, cerebral blood flow regulation and neurovascular homeostasis (Sweeney et al., 2016; Yamazaki and Mukouyama, 2018). In the central nervous system, tight regulation of local cerebral blood flow is essential to match vascular supply to the rapidly changing metabolic demands of neuronal activity. Pericytes contribute to this regulation, although their contractile properties and α-SMA expression vary along the cerebral microvascular tree. In particular, α-SMA-high ensheathing pericytes located at the arteriole–capillary transition exhibit greater contractile capacity and can rapidly regulate vessel diameter, whereas downstream capillary pericytes express substantially lower levels of α-SMA and can contribute to slower modulation of capillary tone and blood flow through distinct contractile and signaling mechanisms (Hartmann et al., 2022; Longden et al., 2023). Thus, the comparatively higher α-SMA expression retained by brain-derived pericytes in our *ex vivo* conditions may be consistent with the specialized involvement of cerebral mural cells in the fine regulation of microvascular perfusion. In contrast, pulmonary pericytes reside within highly specialized capillary networks optimized for gas exchange, vascular permeability control and tissue repair (Yuan et al., 2021; Klouda et al., 2025). The pulmonary circulation constitutes a low-resistance, high-capacitance vascular bed, potentially imposing different requirements on pericyte contractile properties. These distinct physiological and hemodynamic demands may therefore contribute to the differential α-SMA phenotype observed between brain- and lung-derived pericytes.

Importantly, α-SMA (encoded by the ACTA2 gene) expression is not restricted to a stable contractile pericyte phenotype. Recent evidence indicates that α-SMA can also be upregulated during pericyte activation and phenotypic switching. In the lung, pericytes can acquire a smooth muscle-like phenotype characterized by increased α-SMA expression during vascular remodeling, whereas brain pericytes can undergo disease- or inflammatory-associated phenotypic transitions accompanied by changes in smooth muscle actin expression. Thus, the differences in α-SMA observed between brain- and lung-derived pericytes may reflect both tissue-specific mural cell identity and distinct states of pericyte plasticity or activation (Moss et al., 2026; Bohannon et al., 2025).

The distinct phenotypic signature observed in brain-derived pericytes may also be considered in the context of their specialized role in the maturation and maintenance of the blood–brain barrier (BBB). Pericytes are essential components of the neurovascular unit and contribute to BBB development and homeostasis through reciprocal interactions with endothelial cells, astrocytes and the vascular basement membrane. These interactions regulate endothelial junctional organization, vesicular trafficking, basement membrane composition and vascular stability, thereby contributing to the low permeability characteristic of the cerebral microvasculature (Sweeney et al., 2016; Chen et al., 2017). In this context, the comparatively higher expression of RGS5, α-SMA and CD90 observed in brain-derived pericytes may reflect aspects of the specialized mural and mesenchymal phenotype associated with the cerebral vascular niche. RGS5 is strongly associated with mural cell identity and vascular remodeling, whereas α-SMA reflects contractile specialization in specific subsets of cerebral pericytes and may contribute to regulation of microvascular tone (Sweeney et al., 2016; Bohannon et al., 2021). The higher CD90 expression observed in brain-derived cells is also consistent with a distinct mesenchymal and adhesive phenotype, although its specific contribution to BBB function remains to be established. Collectively, these findings suggest that brain-derived pericytes retain a tissue-specific phenotypic signature *ex vivo* that may be related to their specialized functions within the neurovascular unit. Nevertheless, direct functional relationships between these individual markers and BBB maturation or integrity cannot be inferred from the present study and will require validation in appropriate neurovascular models.

The biological relevance of these tissue-specific differences likely extends beyond developmental biology and tissue homeostasis. Increasing evidence indicates that pericytes are active regulators of inflammatory and vascular responses across multiple organs. In the lung, they participate in leukocyte recruitment, cytokine signaling, vascular remodeling and fibrotic responses during chronic inflammatory diseases, pulmonary hypertension and acute lung injury (Hung et al., 2013; Yuan et al., 2019; Yuan et al., 2021; Rayner et al., 2023). Similarly, brain pericytes regulate blood–brain barrier integrity, neuroinflammation and tissue repair following injury (Sweeney et al., 2016; Bohannon et al., 2021; Salinas et al., 2026a). These observations indicate that pericytes are not merely structural vascular cells but active coordinators of tissue-specific inflammatory responses. Despite their phenotypic differences, pericytes from both organs appear to converge on common pathogenic mechanisms during vascular injury. Supporting this concept, recent studies identified pericytes as key mediators of vascular dysfunction during SARS-CoV-2 infection. Preferential viral uptake by pulmonary pericytes promotes vascular damage and increased permeability (Khan et al., 2022), whereas in the brain SARS-CoV-2 induces pericyte-mediated capillary constriction and neurovascular dysfunction (Hirunpattarasilp et al., 2023). Together, these findings reinforce the concept of a lung–brain vascular axis in which tissue-specific pericyte programs converge on shared inflammatory and vascular responses.

The remarkable plasticity of pericytes also has important implications for cancer. In glioblastoma, pericytes have emerged as active regulators of the tumor microenvironment rather than passive vascular support cells. Glioblastoma cells induce profound phenotypic and functional changes in brain pericytes, promoting immunosuppressive programs that facilitate tumor progression (Valdor et al., 2019). Accumulating evidence from our laboratory indicates that chaperone-mediated autophagy (CMA) constitutes a central molecular mechanism governing these transitions. Rather than simply maintaining cellular proteostasis, CMA regulates pericyte homeostasis, preserving their regenerative, tissue-supportive and mesenchymal-associated phenotype (Salinas et al., 2026a). Conversely, inflammatory conditions that reduce CMA activity promote the loss of mesenchymal-associated characteristics together with the acquisition of a more inflammatory and immunogenic phenotype. Notably, several markers analyzed in the present study, including CD90 and CD105, have previously been linked to CMA-dependent maintenance of pericyte identity (Salinas et al., 2026a), suggesting that the differential mesenchymal organization observed between brain- and lung-derived pericytes may reflect differences in the activity or regulation of homeostatic molecular programs. In this context, CMA emerges as a dynamic regulator of pericyte functional state, controlling the balance between regenerative, inflammatory and tumor-supportive phenotypes.

This concept is further supported by recent observations demonstrating that pericytes located at different distances from glioblastoma lesions display distinct immune-regulatory properties, highlighting the existence of specialized pericyte states shaped by local environmental signals (Buizza et al., 2025). Together with our findings, these observations suggest that the enhanced mesenchymal-associated phenotype identified in brain CD146^+^ pericytes, particularly their higher CD90 expression, may influence their susceptibility to tumor-mediated reprogramming. More broadly, they support the concept that pericyte function is determined not only by tissue origin but also by dynamic regulatory mechanisms, such as CMA, that govern transitions between regenerative, inflammatory and tumor-associated states.

Although the present study did not directly address metastatic disease, the comparison between brain- and lung-derived pericytes may also be relevant to organ-specific tumor dissemination. Recent studies have shown that lung cancer stem cells can acquire pericyte-like properties that facilitate brain metastasis by promoting trans-endothelial migration and vascular remodeling (Huang et al., 2023). Likewise, CD146^+^ pericyte-like lung cancer stem cells have been shown to promote tumor angiogenesis through regulation of the VEGF/VEGFR signaling axis (Liu et al., 2026). These observations further support the concept that pericyte-associated programs contribute to the establishment of permissive vascular niches during tumor progression.

Importantly, the comparative characterization of brain- and lung-derived pericytes under standardized *ex vivo* conditions also provides information on the phenotypic properties retained by these cells following isolation and expansion. Given the mesenchymal nature and remarkable plasticity of pericytes, defining this baseline phenotype is relevant for their use as experimental cellular models and provides a framework for subsequent studies addressing tissue-specific responses to pathological stimuli.

A limitation of the present study is that the comparative characterization was performed using primary pericytes expanded *ex vivo*. Direct characterization of freshly isolated pericytes is technically challenging because of the limited number of cells that can be recovered from the microvascular fraction and the further reduction in cellular yield associated with enrichment and purification procedures. The resulting cell numbers are generally insufficient to perform the extensive and repeated phenotypic analyses required in this study. Therefore, *ex vivo* expansion was necessary to obtain sufficient numbers of purified pericytes and to enable a comprehensive comparison of brain- and lung-derived cells under standardized experimental conditions.

Importantly, pericyte subpopulations displaying a phenotype characterized by the co-expression of the conventional pericyte-associated markers analyzed in this study (CD146, PDGFRβ, RGS5, and α-SMA) exhibited enhanced mesenchymal characteristics compared with populations not displaying this combined marker profile. The association between this conventional pericyte-associated marker profile and mesenchymal properties provides evidence that the cultured cells retain phenotypic characteristics associated with pericyte identity following *ex vivo* expansion. Importantly, the absence of this combined marker profile should not be interpreted as evidence of a non-pericyte identity, since pericytes are heterogeneous and the expression of individual markers may vary among pericyte populations. Thus, these findings should be interpreted within the context of the marker panel analyzed in the present study.

Nevertheless, we acknowledge that this finding does not establish complete preservation of the native *in vivo* pericyte state. Previous studies using cultured lung pericytes have shown that these cells retain characteristic pericyte/mesenchymal features and display distinct immunological properties (Hung et al., 2017). At the same time, culture conditions and the loss of the native vascular microenvironment can influence pericyte phenotypic fidelity and transcriptional state (McErlain et al., 2024; Oliveira et al., 2023). Indeed, transcriptomic comparison of cultured mouse brain pericytes at passage 5 with adult pericytes analyzed directly *in vivo* showed that cultured cells constitute a more homogeneous population and display a transcriptional profile more closely resembling embryonic than adult pericytes (Oliveira et al., 2023). Therefore, the differences identified in our study should be interpreted as tissue-of-origin-associated phenotypic properties retained under standardized *ex vivo* conditions rather than as a complete representation of the native *in vivo* phenotype.

Future studies should determine whether these tissue-specific differences are also present within the native vascular microenvironment. Given the limited cellular yield obtained from freshly isolated pericytes, direct tissue-based approaches, including immunofluorescence, immunohistochemistry, RNA *in situ* hybridization, and spatially resolved molecular profiling of brain and lung tissue, including human biopsy specimens when available, would provide valuable complementary validation while preserving the cellular and anatomical context of the native perivascular niche. An additional limitation is that our isolation approach integrates pericytes from different anatomical and microvascular regions within each organ and therefore does not resolve potential region-specific pericyte heterogeneity.

Collectively, our findings support a model in which pericytes retain a conserved mesenchymal-associated core identity while tissue-specific programs determine the organization of this mesenchymal phenotype according to the physiological requirements of each organ. Among the markers analyzed, CD90 emerged as the principal feature distinguishing brain- and lung-derived pericytes, whereas CD44 remained remarkably conserved across tissues. Together with the marked differences observed in canonical pericyte markers such as α-SMA, these findings indicate that tissue specialization is superimposed on a conserved mesenchymal framework. Understanding how developmental origin, local environmental cues and regulatory mechanisms such as chaperone-mediated autophagy coordinate these transitions may ultimately reveal new therapeutic opportunities to modulate pericyte function during tissue regeneration, chronic inflammation, vascular dysfunction and cancer.

## Data Availability

The raw data supporting the conclusions of this article will be made available by the authors, without undue reservation.
