# Supplementary material for: Comparative characterization of lung and brain pericytes reveals tissue-specific marker and mesenchymal signatures
Source: Front Cell Dev Biol. 2026 Oct 30;14:1950106. doi: 10.3389/fcell.2026.1950106 (PMC13760236; doi:10.3389/fcell.2026.1950106)
Supplement: Supplementary file 1 [file Supplementaryfile1.docx]

Supplementary Material

# 1 Supplementary Data

## 1.1 Flow cytometry analysis

Absence of hematopoietic and endothelial cells in the PCs culture was checked by using the following antibodies diluted in FACS Buffer: CD31-PE (Miltenyi Biotec, 27-111-540), CD34-FITC (Miltenyi Biotec, 130-117-775), and CD45-PE (Miltenyi Biotec, 130-110-799). Labelled cells were analyzed by flow cytometry using a BD LSR Fortessa X-20 flow cytometer (BD Bioscience) and data was analyzed with FlowJo software (Ver. 10.0.7, TreeStar). All determinations were performed in at least three separate experiments.

# 2 Supplementary Figures and Tables

## 2.1 Supplementary Figures

**Supplementary Figure 1.** Flow cytometry gating strategy of murine pericytes isolated from brain (PCBs) and lung (PCLTs). Representative multiparametric flow cytometry plots illustrating the sequential gating strategy used to characterize pericyte populations. Cells were first gated based on forward and side scatter to identify the pericyte population, followed by doublet exclusion to select single cells. Viable cells were then identified by negative staining for a viability dye (BV510). From this live cell population, cells were gated based on CD146 expression to evaluate CD146+ and CD146- fractions. To comprehensively characterize the expression of classic pericyte and mesenchymal stem cell markers, two distinct flow cytometry panels were performed where the following antibodies were mixed: Panel 1 consisted of a mixture including antibodies against α-SMA, PDGFRβ, NG2, CD90, CD105, and CD44. Panel 2 consisted of a mixture including antibodies against α-SMA, PDGFRβ, RGS5, CD90, CD105, and CD44. Mesenchymal stem cell markers (CD90, CD105, CD44) are shown sub-gated from the PDGFRβ+ population in Panels 1 and 2; and also from the RGS5+ population in Panel 2. Positive gates for all markers were established according to their corresponding isotype controls.

**Supplementary Figure 2.** Mesenchymal Marker Expression Profile in CD146-Stratified Brain-Derived Pericytes. (**A, B**) Flow cytometry analysis of mesenchymal marker expression (CD90, CD105, and CD44) in primary brain-derived pericytes (PCBs) after magnetic separation into CD146⁺ (**A**) and CD146⁻ (**B**) fractions. Cells were stratified into PDGFRβ⁺ (upper histograms) and RGS5⁺ (lower histograms) subpopulations. Data are expressed as mean fluorescence intensity (MFI). Nonspecific fluorescence was assessed using isotype-matched control antibodies (light grey histograms) compared to specific marker expression (dark grey histograms).

**Supplementary Figure 3.** Mesenchymal Marker Expression Profile in CD146-Stratified Lung-Derived Pericytes. (**A, B**) Flow cytometry analysis of mesenchymal marker expression (CD90, CD105, and CD44) in primary lung pericytes (PCLTs) after magnetic separation into CD146⁺ (**A**) and CD146⁻ (**B**) fractions. Cells were stratified into PDGFRβ⁺ (upper histograms) and RGS5⁺ (lower histograms) subpopulations. Data are expressed as mean fluorescence intensity (MFI). Nonspecific fluorescence was assessed using isotype-matched control antibodies (light grey histograms) compared to specific marker expression (dark grey histograms).

**Supplementary Figure 4.** Absence of endothelial and hematopoietic contamination in brain and lung pericytes. Representative flow cytometry histograms evaluating the purity of isolated pericytes. (**A**) PCBs and (**B**) PCLTs, including both CD146^+^ and CD146^-^ fractions, were analyzed for the expression of endothelial and hematopoietic markers. The lack of positive events in the gated regions confirms that neither PCBs nor PCLTs exhibited contamination with CD31^+^, CD34^+^, or CD45^+^ cells. Histograms are representative of at least three independent experiments.
